# Postoperative results, learning curve, and outcomes of pancreatectomy with arterial resection: a single-center retrospective cohort study on 236 procedures

**DOI:** 10.1097/JS9.0000000000000971

**Published:** 2023-12-11

**Authors:** Niccolò Napoli, Emanuele F. Kauffmann, Carlo Lombardo, Michael Ginesini, Armando Di Dato, Lucrezia Lami, Elena Annunziata, Fabio Vistoli, Daniela Campani, Carla Cappelli, Gabriella Amorese, Ugo Boggi

**Affiliations:** a Division of General and Transplant Surgery; b Division of Pathology, University of Pisa; c Division of Radiology; d Division of Anesthesia and Intensive Care, University of Pisa and Azienda Ospedaliero Universitaria Pisana, Pisa, Italy

**Keywords:** artery (or arterial) resection, learning curve, pancreatic cancer, pancreatic ductal adenocarcinoma, postoperative outcome, vein (or venous) resection

## Abstract

**Background:**

Newer chemotherapy regimens are reviving the role of pancreatectomy with arterial resection (PAR) in locally advanced pancreatic cancer. However, concerns about the early outcomes and learning curve of PAR remain. This study aimed to define the postoperative results and learning curve of PAR and provide preliminary data on oncologic outcomes.

**Materials and methods:**

A single center’s experiences (1993–2023) were retrospectively analyzed to define the postoperative outcomes and learning curve of PAR. Oncologic results were also reported.

**Results:**

During the study period 236 patients underwent PAR. Eighty PAR (33.9%) were performed until 2012, and 156 were performed thereafter (66.1%). Pancreatic cancer was diagnosed histologically in 183 patients (77.5%). Induction therapy was delivered to 18 of these patients (31.0%) in the early experience and to 101 patients (80.8%) in the last decade (*P*<0.0001). The superior mesenteric artery (PAR-SMA), celiac trunk/hepatic artery (PAR-CT/HA), superior mesenteric/portal vein, and inferior vena cava were resected in 95 (40.7%), 138 (59.2%), 189 (80.1%), and 9 (3.8%) patients, respectively. Total gastrectomy was performed in 35 (18.5%) patients. The 30-day mortality rate was 7.2% and 90-day mortality rate was 9.7%. The learning curve for mortality was 106 PAR [16.0 vs. 4.6%; odds ratio, OR=0.25 (0.10–0.67), *P*=0.0055]. Comparison between the PAR-SMA and PAR-CT/HA groups showed no differences in severe postoperative complications (25.3 vs. 20.6%), 90-day mortality (12.6 vs. 7.8%), and median overall survival. Vascular invasion was confirmed in 123 patients (67.2%). The median number (interquartile range) of examined lymph nodes was 60.5 (41.3–83) and rate of R0 resection was 66.1% (121/183). Median overall survival for PAR was 20.9 (12.5–42.8) months, for PAR-SMA was 20.2 (14.4–44) months, and for PAR-CT/HA was 20.2 (11.4–42.7). Long-term prognosis improved by study decade [1993–2002: 12.0 (5.4–25.9) months, 2003–2012: 15.1 (9.8–23.4) months, and 2013–present: 26.2 (14.3–51.5) months; *P*<0.0001].

**Conclusions:**

In recent times, PAR is associated with improved outcomes despite a steep learning curve. Pancreatic surgeons should be prepared to face the technical challenge posed by PAR.

## Introduction

Highlights

- Upon completion of the learning curve (106 procedures), 90-day mortality was 4.6%.
- R0 resection was achieved in greater than 65% of the patients with pancreatic cancer.
- Long-term prognosis improved overtime.
- Prognosis was not affected by which artery was resected.
- Diarrhea marked the early postoperative period, but could be managed.

Pancreatic cancer is one of the most lethal malignancies. In the United States although it accounts for only 3% of all new cancer diagnoses, it causes 8% of cancer-related deaths and is the fourth leading cause of cancer death in both males and females^1^. The poor prognosis of pancreatic ductal adenocarcinoma (PDAC) is mostly caused by an aggressive tumor biology, characterized by early development of distant metastases^2^; resistance to chemotherapy^3^, radiotherapy^4^, and immunotherapy^5^, and rarity of actionable gene mutations^6^.

Despite this grim situation, multimodality treatment can result in the long-term survival of select patients^7^. PDAC may have a predominantly localized growth pattern in about one-third of patients^8^. When there is no evidence of distance metastasis, tumor markers are low or significantly decreased, and a patient’s conditions are excellent after a period of oncological treatment, surgery may become an option based on the new concept of prognosis-based resectability^9^. However, in clinical practice, relevant anatomic tumor features, such as arterial involvement, are often considered and can independently lead to the judgement of unresectability. Dealing with arterial resections makes surgery more complex and risky; however, in this modern chemotherapy and chemo-radiation era, no sound evidence supports the association between radiologic arterial involvement and worse prognosis^9^.

Unfortunately, information about pancreatectomy with artery resection (PAR) is sparse and low in quality. According to the IDEAL framework for the evaluation of surgical innovation^10^, PAR is graded as stage 1 (idea) or stage 2a (development). Therefore, oncologic outcomes (IDEAL stage 3; assessment) that are key to justifying such extended procedures cannot be fully addressed at the present time. Practically, the feasibility and safety of PAR are still being determined.

Our group pioneered pancreatectomy with vascular resection, with the first procedures performed in the early 1980s. After experience with vein resections, arteries were also resected^11–15^. Currently, following preoperative oncological treatments, PAR is one of the treatment options for locally advanced PDAC^16^. Most concerns are related to the resection and reconstruction of the superior mesenteric artery (PAR-SMA).

In this study, we aimed to define the postoperative results and learning curve of PAR. Preliminary data on oncologic outcomes were also provided.

## Methods

This study retrospectively analyzed a prospectively maintained database including all pancreatic resections performed at a single center from 1 January 1993 to 19 May 2023. This study was approved by the Institutional Institutional Ethics Board of the University of Pisa (21060_Boggi), registered retrospectively on Research Registry (study registration ID: researchregistry9687), and performed according to the principles of the Declaration of Helsinki^17^, Strengthening the Reporting of Observational Studies in Epidemiology guidelines on reporting on observational studies^18^, and Strengthening the Reporting of Cohort Studies in Surgery criteria^19^.

### Definition of outcome metrics

Postoperative mortality was defined as any death occurring within 90 days after surgery or during hospital stay, if longer than 90 days.

Pancreas-specific complications (i.e. postoperative pancreatic fistula, delayed gastric emptying, and postpancreatectomy aemorrhage) were defined according to the International Study Group on Pancreatic Surgery (ISGPS)^20–22^.

The Clavien–Dindo classification was used to assess the severity of postoperative complications. Severe complications were those graded ≥3^23^. The comprehensive complication index was employed to define the overall burden of surgical complications in each patient^24^.

### Selection criteria for PAR

Patients requiring arterial repair following surgical misadventure were not included. No other general exclusion criteria were applied, and all tumor types were considered.

The selection criteria for PAR are detailed in two previous studies^12,13^. After the year 2000, all surgical candidates were approved during multidisciplinary discussions. All patients were staged by total-body contrast-enhanced computed tomography, and their CEA, Ca 19.9, and Ca 125 levels were assayed. Additional diagnostic modalities were used according to individual needs as required. Excluding aortic involvement, if a strategy for resection and reconstruction of the involved vessel(s) could be defined, no degree of vascular involvement and/or type of involved vessel was considered an absolute contraindication to surgery.

Until 2012 (i.e. before the availability of effective multiagent chemotherapy) patients were mostly selected based on the absence of distant metastasis, patient performance status (ECOG 0-1), and technical feasibility. Some of these patients received preoperative oncological treatments, but these treatments were not mandatory and typically not performed as neoadjuvant treatments. Patients were mostly referred for possible tumor resection due lack of tumor progression, often after several months without oncological treatments. In suitable candidates, presence of occult metastasis was further defined by laparoscopy before laparotomy was performed.

After 2012, preoperative oncological treatments became mandatory before the evaluation process for resection could begin. Lack of tumor progression on contrast-enhanced computed tomography scans performed within 4 weeks of surgery was required in all patients. Tumor regression/stability was a marker of response to oncological treatments. In patients with high Ca 19.9 levels, a decrease of ≥50% of basal values was also required. In patients with low baseline Ca 19.9 levels, low CEA and Ca 125 levels were required. MRI was used selectively, mostly to characterize liver lesions with indeterminate findings on computed tomography images.

All patients were comprehensively informed about the innovative nature of PAR, as well as the alternative treatment modalities. Each patient signed an informed consent.

### Study groups

Study groups included PAR, PAR-SMA, and PAR with resection and reconstruction of the celiac trunk/hepatic artery (PAR-CT/HA). Subgroup analysis was performed for patients undergoing PAR-SMA and PAR-CT/HA concurrently.

### Surgical technique

Surgical technique of PAR was described in a previous study^13^. No support was provided by vascular surgeons. Operating surgeons had received training in vascular surgery and were in charge of an abdominal organ transplantation program. The type of vascular resection/reconstruction was classified according to the ISGPS^25^. All dissections were performed *in situ*, with intact patient circulation. In patients with portomesenteric vein occlusion, dissection was carefully performed by preserving the collateral circulation until the appropriate time for resection and reconstruction. Transitory mesenterico-portal or mesenterico-caval shunt were selectively employed.

The point of no return was trespassed if safe vascular reconstruction (when needed) could be ensured. The use of vascular prosthesis was avoided. In type 4 resection/reconstruction, autologous grafts were preferred. The internal jugular vein was used for most venous reconstructions and the greater saphenous vein for most arterial reconstructions. The greater saphenous vein was also used for vein reconstructions, either as a vascular patch or spiral graft matching the size of the portomesenteric vein. Alternative options for arterial reconstructions included clockwise rotation of the splenic artery and harvesting of the internal iliac artery.

When no autologous graft was available or suitable, vascular grafts from deceased donors were also used. These grafts were not cryopreserved but were obtained from our abdominal organ transplantation program after a short of storage period (<2 weeks) at 4°C in the Terasaki solution or in the same solution used for organ preservation. These grafts were used based on AB0 compatibility. In type 2 resection/reconstruction, the peritoneum was also used^13^.

More details about our PAR approach are presented in the Supplementary Digital content (SDC, Videos 1–4, Supplemental Digital Content 1, http://links.lww.com/JS9/B554, Supplemental Digital Content 2, http://links.lww.com/JS9/B555, Supplemental Digital Content 3, http://links.lww.com/JS9/B556, Supplemental Digital Content 4, http://links.lww.com/JS9/B557).

### Postoperative surveillance and anticoagulation

All patients were admitted to the ICU based on a protocol. Lactate levels were assessed every 6 h for the first 48 h, and liver perfusion was confirmed 12 h after surgery using Doppler ultrasonography. Lactate dehydrogenase, aspartate transaminase, and alanine were assayed at the end of surgery and every 12 h for the next 48 h after surgery. In case of high or increasing lactate and/or enzyme levels, as well as unexplained hemodynamic instability and/or acute renal failure, a contrast-enhanced computed tomography scan was urgently performed. In the absence of clinical and/or laboratory suspicion of visceral ischemia, a contrast-enhanced computed tomography scan was performed on postoperative day 7 based on a protocol.

Details of the anticoagulation strategy was described in a previous study^13^. Patients received an intraoperative bolus of unfractionated heparin (60–80 IU/kg body weight) and were subsequently maintained for 4 weeks under a standard antithrombotic prophylaxis regimen based on low-molecular-weight heparin. No chronic anticoagulant or antiaggregant therapy was administered independently for vascular reconstructions.

### Specimen histology

Histology was performed by dedicated pathologists according to contemporary standards and recommendations. After 1 January 2008, margins of tumors were assessed circumferentially using whole-mount histological slides^15^. Microscopic tumor residual (R1) was defined based on the 1 mm rule^26^.

The tumor, nodes, and metastases stage was defined according to the American Joint Committee on Cancer classification (8th edition).

### Follow-up

Following the introduction of gemcitabine, adjuvant chemotherapy has been recommended for all patients with PDAC, irrespective of whether they had already received preoperative treatments.

During the first year after resection, all patients visited our institution every 3 months, with the results of a thoraco-abdominal computed tomography scan and an assay of tumor markers (CEA, Ca 19.9, and Ca 125). Patients were specifically assessed more frequently to manage intestinal function. Subsequently, patients who lived far from Pisa city were managed in collaboration with their local oncologists. All patients underwent an assay of tumor markers every 3 months and a thoraco-abdominal computed tomography scan every 6 months for at least 5 years.

### Postoperative diarrhea and nutritional status

Occurrence of diarrhea and nutritional status were assessed in a subgroup of patients before (t0), 30 days (t1), 90 days (t2), and 6 months (t3) after surgery. The nutritional status was assessed using the geriatric nutritional risk index^27^.

### Statistical analysis

Categorical variables are summarized as frequencies, percentages, and rates. Continuous variables are expressed as mean±SD or as median and interquartile range [IQR], if normally or non-normally distributed, respectively. Normality distribution was assessed using the Shapiro–Wilk test.

The Pearson χ^2^and Fisher’s exact tests (if group population is <5) were used to compare categorical variables between different groups. The Wilcoxon and Kruskal–Wallis tests were preferred for comparing continuous variables.

Time-to-event endpoint (overall survival) was estimated using the nonparametric Kaplan–Meier method. The Log-rank test was used to compare survival between different groups.

The learning curve process was assessed using the cumulative sum method (CUSUM). The values of 0 for success and 1 for the failure were used for the categorical variables. Their respective values were used for continuous variables. Based on the CUSUM analysis, the overall population was divided into two or more groups (groups A, B and C, if necessary). Student’s *t*-test was used to compare continuous variables, while the χ^2^ and Fisher’s exact tests were used to compare categorical variables. The odds ratio (OR) and ‘t’ were used as an estimation of the effect size for categorical variables and continuous variables, respectively.

Logistic regression analyses, both univariate and multivariate, were employed to assess the potential influence of covariates on the incidence of severe postoperative complications and mortality following PAR-SMA. Similarly, a univariate and multivariate Cox regression analysis was used to assess the impact of potential confounders on long-term survival after PAR-SMA. Lastly, using the *obsSens* package in R (version 4.3.0, R Foundation for Statistical Computing), sensitivity analysis was performed to determine whether any potential categorical and continuous variables were not assessed.

All statistical analyses were performed using JMP Pro 16.0.0 software package for Mac (Copyright SAS Institute Inc., SAS campus Drive) and R Package, R Core Team (2014): A language and Environment for Statistical Computing (R Foundation for Statistical Computing) version 4.3.0(2023-04-21) using the Rstudio by Posit.

## Results

**Figure 1:**
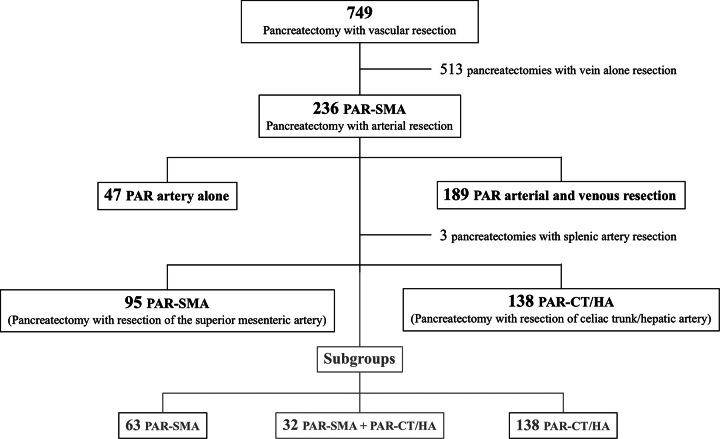
Study flow diagram.

Figure 1 reports the study flowchart. Eighty PAR (33.9%) were performed until 2012 and 156 PAR (66.1%) thereafter (*P*<0.0002). Before 2012, 24 patients (30.0%) and in the last decade, 118 patients (75.6%) underwent induction therapy (*P*<0.0001). Equivalent figures for PDAC were 31.0% (18/58) and 80.8% (101/125), respectively (*P*<0.0001).

PAR consisted of only artery resection in 47 patients (19.9%) and a combined arterial and venous resection in the remaining 189 patients (80.1%). In 204 patients, a single artery was resected (86.4%), while in 32 patients two arteries were resected (13.6%). The SMA was resected and reconstructed in 95 patients (40.3%), either alone (4; 4.2%) or in combination with the superior mesenteric/portal vein (91; 95.8%). The celiac trunk/hepatic artery was resected in 170 patients (72.0%), either alone (44; 25.8%) or in combination with a vein (126; 74.1%). In three patients the splenic artery was resected and reconstructed (1.3%). Overall, 268 arteries were resected. The superior mesenteric/portal vein was resected in 189 patients (80.1%) and the inferior vena cava in nine patients (3.8%). Overall, 466 vessels in 236 patients were resected.

Median operative time was 610 (530–700) min for all PAR, 450 (379–575) min for only artery resection and 635 (560–720) min for combined arterial and venous resection (*P*<0.0001).

Total gastrectomy was required in 35 patients (18.5%) who underwent combined arterial and venous resection and in none among those requiring isolated arterial resection. Table 1 provides a summary of the remaining general characteristics of the study population, including a comparison between artery resection alone and combined arterial and venous resection. No patient deemed technically resectable on preoperative imaging studies was deemed unresectable intraoperatively in the absence of distant metastasis. No macroscopic tumor residue remained.

**Table 1:** General characteristics, operative details, main postoperative outcomes and histology of 236 PAR.

	PAR (*n*=236)	Artery alone (*n*=47)	Artery and vein (*n*=189)	*P* ^a^
Baseline characteristics				
Age, median (IQR), years	63.3 (55.6–70.4)	63.7 (56.7–72.6)	63.2 (54.8–70.2)	0.544
Male sex, *n* (%)	124 (47.5)	25 (53.2)	99 (52.4)	0.921
BMI, median (IQR), kg/m^2^	23.5 (21.3–25.7)	23.4 (21.3–25.3)	23.5 (21.3–25.7)	0.942
ASA score, median (IQR)	3 (2–3)	3 (2–3)	3 (2–3)	0.715
Diabetes, *n* (%)	62 (26.3)	13 (27.7)	49 (25.9)	0.809
Cardiac disease, *n* (%)	23 (9.7)	4 (8.5)	19 (10.1)	1.000
Chronic obstructive pulmonary disease, *n* (%)	12 (5.1)	7 (14.9)	5 (2.7)	0.0029
Previous abdominal surgery, *n* (%)	94 (39.8)	19 (40.4)	75 (39.7)	0.926
Complaints				
Any symptom, *n* (%)	195 (82.6)	35 (74.5)	160 (84.7)	0.0990
Obstructive jaundice, *n* (%)	75 (31.8)	11 (23.4)	64 (33.9)	0.168
Abdominal/back pain, *n* (%)	125 (53.0)	24 (51.1)	101 (53.4)	0.7703
Weight loss, *n* (%)	63 (26.7)	11 (23.4)	52 (27.5)	0.569
Digestive issues, *n* (%)	28 (11.9)	4 (8.5)	24 (12.7)	0.614
Tumor site^b^				
Head/uncinate process, *n* (%)	171 (72.5)	16 (34)	155 (82)	<0.0001
Body, *n* (%)	65 (27.5)	31 (66)	34 (18)	<0.0001
Surgical approach				
Open, *n* (%)	229 (97)	43 (91.5)	186 (98.4)	0.0307
Minimally invasive, *n* (%)	7 (3)	4 (8.5)	3 (1.6)	0.0307
Type of pancreatic resection				
Pancreaticoduodenectomy, *n* (%)	35 (14.8)	10 (21.3)	25 (13.2)	0.165
Total pancreatectomy, *n* (%)	165 (69.9)	8 (17.0)	157 (83.1)	<0.0001
Distal pancreatectomy, *n* (%)	36 (15.3)	29 (61.7)	7 (3.7)	<0.0001
Reconstruction of the superior mesenteric artery				
Direct reconstruction, *n* (%)	53 (55.8)	2 (50)	51 (56.0)	1.000
Interposition graft, *n* (%)	21 (22.1)	2 (50)	19 (20.9)	0.211
Splenic artery rotation, *n* (%)	21 (22.1)	0 (0)	21 (23.1)	0.572
Reconstruction of the celiac trunk/hepatic artery				
No recontruction, *n* (%)	28 (16.5)	20 (45.5)	8 (6.4)	<0.0001
Direct reconstruction, *n* (%)	37 (21.8)	6 (13.6)	31 (24.6)	0.144
Interposition graft, *n* (%)	81 (47.7)	18 (40.9)	63 (50)	0.299
Splenic artery rotation, *n* (%)	24 (14.1)	0 (0)	24 (19.1)	0.0007
Reconstruction of the superior mesenteric/portal vein				
Type 1, *n* (%)	1 (0.5)	NA	1 (0.5)	NA
Type 2, *n* (%)	5 (2.6)	NA	5 (2.7)	NA
Type 3, *n* (%)	144 (76.2)	NA	14 (76.2)	NA
Type 4, *n* (%)	39 (20.6)	NA	39 (20.6)	NA
Main postoperative outcomes				
Postoperative mortality, *n* (%)	23 (9.7)	3 (6.4)	20 (10.6)	0.583
Severe postoperative complications	39 (16.5)	7 (14.9)	32 (16.9)	0.736
Failure-to-rescue, *n* (%)	23 (58.9)	3 (42.9)	20 (62.5)	0.415
Hospital readmission, *n* (%)	16 (6.8)	2 (4.3)	14 (7.4)	0.745
Repeat surgery, *n* (%)	21 (8.9)	5 (10.6)	16 (8.5)	0.578
Interventional radiology procedures, *n* (%)	18 (7.6)	3 (6.4)	15 (7.9)	1.000
Interventional endoscopic procedures, *n* (%)	5 (2.1)	0 (0)	5 (2.7)	0.586
Comprehensive complication index, median (IQR)	22.6 (8.7–36.2)	20.9 (0–30.8)	29.6 (8.7–36.2)	0.176
Patients receiving blood transfusions, *n* (%)	95 (40.3)	15 (31.9)	80 (42.3)	0.193
Number of blood transfusions per patient, median (IQR)	0 (0–2)	0 (0–2)	0 (0–2)	0.158
Grade B/C POPF^c^	10 (4.2%)	6 (15.4%)	4 (12.5%)	1.000
Grade B/C DGE	55 (23.3%)	7 (14.9%)	48 (25.4%)	0.128
Grade B/C PPH	48 (20.3%)	7 (14.9%)	41 (21.7%)	0.300
Bile leak, *n* (%)	1 (0.4)	0 (0)	1 (0.5)	1.000
Chyle leak, *n* (%)	5 (2.1)	1 (2.1)	4 (2.1)	1.000
Intestinal leak, *n* (%)	8 (3.4)	2 (4.3)	6 (3.2)	0.661
Intestinal ischemia, *n* (%)	7 (3.0)	0 (0)	7 (3.7)	0.350
Hepatic ischemia, *n* (%)	8 (3.4)	2 (4.3)	6 (3.2)	0.661
Vascular thrombosis, *n* (%)	13 (5.5)	2 (4.3)	11 (5.8)	1.000
Vein thrombosis, *n* (%)	7 (3.0)	1 (2.1)	6 (3.2)	1.000
Artery thrombosis, *n* (%)	6 (2.5)	1 (2.1)	5 (2.7)	1.000
Histology				
Malignant tumor, *n* (%)	233 (98.7)	45 (95.7)	188 (99.5)	0.102
Pancreatic ductal adenocarcinoma, *n* (%)	183 (77.5)	31 (66.0)	152 (80.4)	0.0493
Malignant IPMN, *n* (%)	24 (10.2)	5 (10.6)	19 (10.1)	1.000
Cholangiocarcinoma, *n* (%)	6 (2.5)	2 (4.3)	4 (2.1)	0.343
Pancreatic neuroendocrine tumor/carcinoma, *n* (%)	5 (2.1)	0 (0)	5 (2.7)	0.586
Sarcoma, *n* (%)	4 (1.7)	1 (2.1)	3 (1.6)	1.000
Duodenal adenocarcinoma, *n* (%)	2 (0.8)	1 (2.1)	1 (0.5)	0.359
Pancreatic metastasis, *n* (%)	2 (0.8)	2 (4.3)	0 (0)	0.0390
Other malignant tumor histology, *n* (%)	7 (3.0)	3 (6.4)	4 (2.1)	0.144
Number of examined lymph nodes, median (IQR)	59 (41–80.8)	37 (24–57)	63 (46.3–88)	<0.0001
Further pathology data for pancreatic ductal adenocarcinoma				
T stage				
T1, *n* (%)	10 (5.5)	2 (6.5)	8 (5.3)	0.678
T2, *n* (%)	67 (36.6)	11 (35.5)	56 (36.8)	0.886
T3, *n* (%)	34 (18.6)	5 (16.1)	29 (19.1)	0.805
T4, *n* (%)	72 (39.3)	13 (41.9)	59 (38.8)	0.746
N stage				
N0, *n* (%)	28 (15.3)	8 (25.8)	20 (13.2)	0.0746
N1, *n* (%)	72 (39.3)	13 (41.9)	59 (38.8)	0.841
N2, *n* (%)	83 (45.4)	10 (32.3)	73 (48.0)	0.108
R0 margin status	121 (66.1)	22 (71.0)	99 (65.1)	0.532
Arterial invasion, *n* (%)	72 (39.3)	13 (41.9)	59 (38.8)	0.746
Length of arterial invasion, median (IQR), mm	10 (6.3–15)	14 (6.3–19.5)	10 (6.3–15)	0.674
Vein invasion, *n* (%)	90 (59.2)	—	90 (59.2)	NA
Length of vein invasion, median (IQR), mm	15 (10–25)	—	15 (10–25)	NA

a Comparison of artery alone versus artery and vein.

b Head/uncinate process tumor category includes also periampullary tumors.

c Relative to partial pancreatectomy.

ASA, American Society of Anesthetists Physical Status; DGE, delayed gastric emptying; IPMN, intraductal papillary mucinuous neoplasm; POPF, postoperative pancreatic fistula; PPH, postpancreatectomy hemorrhage.

**Figure 2:**
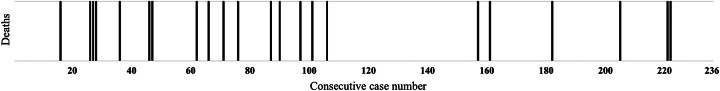
Ninety-day postoperative mortality timeline in 236 pancreatectomy with arterial resection.

The 30-day and 90-day mortalities were 7.2% (17/236) and 9.7% (23/ 236), respectively. The 30-day and 90-day mortalities for artery resection alone were 4.3 and 6.4%, respectively. Equivalent results for combined arterial and venous resections were 7.9 and 10.6%, respectively. Deaths mostly occurred within the first 106 PAR (16.0%) surgeries. Thereafter, six deaths were recorded after 130 PAR (4.6%). A consecutive series of 49 PAR without mortality was achieved between case 106 and 155 (Fig. 2).

The leading causes for mortality were intestinal ischemia (5/236; 2.1%), respiratory distress syndrome (5/236; 2.1%), liver ischemia/failure (3/236; 1.2%), bleeding (3/236; 1.2%), sepsis (3/236; 1.2%), intestinal perforation (2/236; 0.8%), grade C postoperative pancreatic fistula plus intestinal perforation (1/236; 0.4%), and pancreatitis plus superior mesenteric/portal vein thrombosis (1/236; 0.4%).

### Feasibility and safety of laparoscopic and robotic surgery in PAR

Seven PAR (2.9%) were performed using a minimally invasive technique (laparoscopy: 2, 0.8%; robotic-assistance: 5, 2.1%). In five patients, arterial involvement was an incidental finding during surgery. The decision to keep pursuing the minimally invasive surgery was based on judgment of feasibility without undue risk to the patients. There was no conversion to open surgery. Five patients (71.4%) suffered mild postoperative complications (i.e. ≤grade 2), and no patient died.

### Resection and reconstruction of the superior mesenteric artery

PAR-SMA was performed in 95 patients (40.2%). A type 3 reconstruction was employed in 53 patients (55.8%) and type 4 in 21 patients (22.1%). In the remaining 21 patients, the splenic artery was rotated. In 78 patients (82.1%) a single branch of the SMA was reconstructed. Two branches were reconstructed in 12 patients (12.6%) and three branches in five patients (5.3%).

Compared with the 138 PAR-CT/HA, PAR-SMA was associated with more frequent use of total pancreatectomy [86/95 (90.5%) vs. 79/141 (56.0%); *P*<0.0001], with vein resection [91/95 (95.8%) vs. 98/141 (69.5%); *P*<0.0001], inferior vena cava resection [7/95 (7.4%) vs. 2/141 (1.4%); *P*=0.0325], spleen and splenic vessel resection [28/95 (31.8%) vs. 12/141 (10.6%); *P*=0.0002], shorter median length of hospital stay [19 (14–27) vs. 22 (19–30) days; *P*=0.0112] but longer median length of ICU stay [3 (1–6) vs. 2.0 (1.0–4.3) days; *P*=0.0451], and more frequent hospital readmissions [11 (11.6%) vs. 5 (3.6%); *P*=0.0315]. PAR-SMA was performed exclusively through an open approach [95/95 (100%) vs. 134/141 (95.0%); *P*=0.0436]. Operative time was slightly longer in PAR-SMA than in PAR-CT/HA [623 (540–720) vs. 600.0 (492.5–695.0) min; *P*=0.0608], but the difference was not statistically significant.

**Figure 3:**
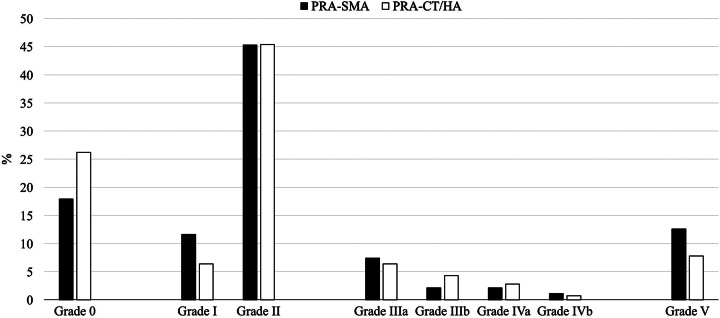
Incidence and severity of postoperative complications following superior mesenteric artery (PAR-SMA) (black bars) and celiac trunk/hepatic artery (PAR-CT/HA) (white bars).

Following PAR-SMA, 17 patients had an uneventful postoperative course (17.9%), 11 (11.6%) developed grade 1 complications, and 43 (45.3%) developed grade 2 complications. Severe complications occurred in 24 patients (grade 3a: 7, 7.4%; grade 3b: 2, 2.1%; grade 4a: 2, 2.1%; grade 4b: 1, 1.0%; and grade 5: 12, 12.6%). Equivalent results for PAR-CT/HA were 9 (6.4%), 6 (4.3%), 4 (2.8%), 1 (0.7%), 11 (7.8%), respectively. No significant difference in the incidence and severity of postoperative complications was observed between PAR-SMA and PAR-CT/HA (Fig. 3). Similarly, no difference in median values of the comprehensive complication index [22.6 (0–36.2) vs. 29.6 (8.7–36.2); *P*=0.173], failure to rescue [10 (31.3%) vs. 8 (20.5%); *P*=0.301], and postoperative mortality [12 (12.6%) vs. 11 (7.8%); *P*=0.220] was observed. Further details regarding the type of postoperative complications and need for invasive management are summarized in Table 2.

**Table 2:** Postoperative complications, invasive treatments, and other relevant outcome metrics in PAR-SMA and PAR-CT/HA.

	PAR-SMA	PAR-CT/HA	*P*
Complications			
Grade B/C POPF	1 (11.1%)	9 (14.5%)	1.000
Grade B/C DGE	24 (25.3%)	31 (22.0%)	0.559
Grade B/C PPH	25 (26.3%)	23 (16.3%)	0.0612
Bile leak, *n* (%)	1 (1.1)	0 (0)	0.403
Chyle leak, *n* (%)	1 (1.1)	4 (2.8)	0.651
Intestinal leak, *n* (%)	3 (3.2)	5 (3.6)	1.000
Intestinal ischemia, *n* (%)	4 (4.2)	3 (2.1)	0.444
Hepatic ischemia, *n* (%)	4 (4.2)	4 (2.8)	0.717
Vein thrombosis, *n* (%)	3 (3.2)	4 (2.8)	1.000
Artery thrombosis, *n* (%)	4 (4.2)	2 (1.4)	0.223
Invasive management of complications			
Percutaneous catheter drainage, *n* (%)	9 (9.5)	9 (6.4)	0.380
Interventional endoscopy procedure, *n* (%)	4 (4.2)	1 (0.7)	0.161
Repeat surgery, *n* (%)	7 (7.4)	14 (9.9)	0.498
Repeat surgery for bleeding, *n* (%)	1 (1.1)	5 (3.6)	0.406
Repeat surgery for vascular thrombosis, *n* (%)	4 (4.2)	2 (1.4)	0.223

### The learning curve of PAR

**Figure 4:**
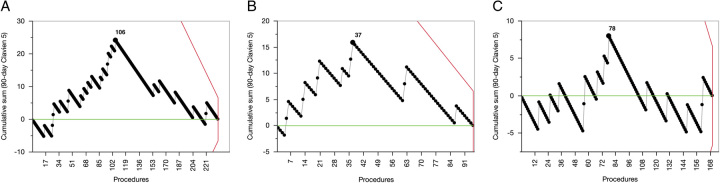
Learning curve for postoperative mortality. A: All PAR; B: PAR-SMA; C: PAR-CT/HA.

Table 3 provides details about the learning curve of PAR. The learning curve of PAR was completed for severe postoperative complications following 115 procedures, for both comprehensive complication index and postoperative mortality following 106 procedures (Fig. 4), for length of postoperative hospital stay following 91 procedures, and for the need to proceed to total gastrectomy following 90 procedures.

**Table 3:** Learning curve of PAR.

Outcome	Learning curve	Group A	Group B	Group C	OR (A/B)	*P*	OR (A/C)	*P*
Severe postoperative complications, *n* (%)								
PRA	115	34/115 (29.6)	19/121 (15.7)	—	2.25 (1.20-4.24)	0.0118	—	—
PRA-SMA	38–74	13/38 (34.2)	9/36 (25.0)	2/21 (9.5%)	1.56 (0.57–4.28)	0.388	4.94 (0.99–24.56)	0.0509
PRA-CT/HA	102	25/102 (24.5)	11/68 (16.2)	—	1.68 (0.77–3.70)	0.195	—	—
Comprehensive complication index, median (IQR)								
PRA	106–182	22.6 (0–39.7)	29.6 (20.9–33.7)	20.9 (6.5–31.1)	1.13 (−3.54–13.09)	0.259	1.44 (−2.50–16.00)	0.152
PRA-SMA	38	29.6 (0–71.3)	29.6 (10.5–33.6)	—	1.51 (−2.96–21.82)	0.134	—	—
PRA-CT/HA	130	20.9 (0–30.8)	22.6 (8.7–36.2)	—	0.76 (−5.79–12.99)	0.451	—	—
Postoperative mortality								
PRA	106	17/106 (16.0%)	6/130 (4.6%)	—	3.95 (1.50–10.41)	0.0055	—	—
PRA-SMA	37	9/37 (24.3%)	3/58 (5.2%)	—	5.89 (1.48-23.51)	0.0120	—	—
PRA-CT/HA	78	8/78 (10.3%)	5/92 (5.4%)	—	1.99 (0.62-6.35)	0.246	—	—
Length of postoperative stay, median (IQR)								
PRA	91	23 (16–30)	20 (14.5–27)	—	2.40 (0.74-7.56)	0.0173	—	—
PRA-SMA	32	22.5 (13–30.8)	22 (20–27)	—	0.75 (-3.20-7.12)	0.452	—	—
PRA-CT/HA	45 – 143	23 (17–30.5)	19.5 (14.8–27)	20 (12–27)	2.20 (0.51–9.65)	0.0295	2.26 (0.89–13.24)	0.0252
Total gastrectomy, *n* (%)								
PRA	90	30/90 (33.3)	7/146 (4.8)	—	9.93 (4.13–23.86)	<0.0001	—	—
PRA-SMA	32	15/32 (46.9)	0/63 (0)	—	904861432 (0–NA)	0.996	—	—
PRA-CT/HA	60	20/60 (33.3)	8/110 (7.3)	—	6.37 (2.60–15.64)	<0.0001	—	—

### Diarrhea and nutritional status

Initially, all patients suffered diarrhea and had impaired nutritional status. Diarrhea and nutritional statuses progressively improved over the first six postoperative months (Tables 4 and 5).

**Table 4:** Bowel function and nutritional status in PAR, PAR-SMA, and PAR-CT/HA.

	t0	t1	t2	t3	*P* (ANOVA)
Steatorrhea (daily bowel movements, mean±SD)					
PAR	0.13±0.32	3.36±0.32	2±0.32	1.13±0.39	<0.0001
PAR-SMA	0±0.64	1.33±0.64	1.33±0.64	0±1.11	0.40
PAR-CT/HA	0.16±0.34	3.68±0.34	2.11±0.34	1.21±0.40	<0.0001
Diarrhea (daily bowel movements, mean±SD)					
PAR	0.86±0.55	4.09±0.55	3.32±0.55	2.13±0.66	0.0005
PAR-SMA	0±0.54	1.33±0.54	2.33±0.54	0±0.94	0.09
PAR-CT/HA	1±0.60	4.53±0.60	3.47±0.60	2.29±0.70	0.0008
Weight (Kg, mean±SD)					
PAR	66.43±2.38	58±2.38	56.81±2.38	58.13±2.82	0.02
PAR-SMA	75±12.68	73±12.68	73±12.68	86±17.93	0.93
PAR-CT/HA	65.53±2.13	56.42±2.13	55.11±2.13	56.14±2.48	0.003
NRI (points, mean±SD)					
PAR	96.29±2.51	85.43±2.57	86.37±2.51	93.83±2.82	0.007
PAR-SMA	107±30.09	64.9±21.28	68.2±21.28	98.5±30.09	0.65
PAR-CT/HA	95.69±1.99	87.99±2.11	88.51±2.05	93.5±2.26	0.02

NRI, nutritional risk index; t0, before surgery; t1, postoperative day 30; t2, postoperative day 39; t3, postoperative day 180.

**Table 5:** Pairwise comparison of intestinal function between follow-up times based on treatment subgroup (PAR, PAR-SMA, and PAR-CT/HA).

	Steatorrhea			Diarrhea			Weight			NRI		
	Difference	SE	*P*	Difference	SE	*P*	Difference	SE	*P*	Difference	SE	*P*
PAR												
t1–t0	3.23	0.45	<0.0001	3.23	0.77	0.0004	−8.43	3.36	0.0674	−10.86	3.59	0.0182
t2–t0	1.86	0.45	0.0005	2.45	0.77	0.0112	−9.61	3.36	0.0277	−9.92	3.54	0.0331
t3–t0	1.00	0.50	0.203	1.27	0.86	0.454	−8.30	3.69	0.119	−2.46	3.77	0.915
t2–t1	−1.36	0.45	0.0180	−0.77	0.77	0.749	−1.19	3.36	0.985	0.95	3.59	0.994
t3–t2	−0.87	0.50	0.318	−1.18	0.86	0.515	1.32	3.69	0.984	7.46	3.77	0.207
PAR-SMA												
t1–t0	1.33	0.90	0.503	1.33	0.77	0.386	−2.00	17.93	1.000	−42.10	36.85	0.707
t2–t0	1.33	0.90	0.503	2.33	0.77	0.0829	−2.00	17.93	1.000	−38.80	36.85	0.745
t3–t0	0.00	1.28	1.00	0.00	1.09	1.000	11.00	21.95	0.954	−8.50	42.55	0.996
t2–t1	0.00	0.90	1.00	1.00	0.77	0.596	0.000	17.93	1.000	3.30	30.09	0.999
t3–t2	−1.33	1.28	0.732	−2.33	1.09	0.2409	−13.00	21.96	0.928	30.30	36.85	0.843
PAR-CT/HA												
t1–t0	3.53	0.48	<0.0001	3.53	0.85	0.0006	−9.11	3.01	0.0180	−7.70	2.90	0.0485
t2–t0	1.95	0.48	0.0008	2.47	0.85	0.0252	−10.42	3.00	0.005	−7.18	2.86	0.0676
t3–t0	1.06	0.52	0.194	1.29	0.93	0.510	−9.38	3.27	0.0272	−2.19	3.01	0.885
t2–t1	−1.58	0.48	0.0091	−1.05	0.85	0.607	−1.32	3.01	0.972	0.52	2.94	0.998
t3–t2	−0.89	0.52	0.333	−1.19	0.93	0.576	1.04	3.27	0.999	4.99	3.05	0.366

NRI, nutritional risk index; t0, before surgery; t1, postoperative day 30; t2, postoperative day 39; t3, postoperative day 180-

### Histology of PAR

The histological results of PAR are presented in Table 6. Only three cases of nonmalignant histological results (1.3%) were observed in a series that included cases operated in the 1990s and early 2000s. In the context of accurate specimen pathology [median 97 number of examined lymph nodes: 59 (41.0–80.8)], no patient underwent R2 resection, and 70% of patients underwent R0 99 resection. Vascular infiltration was confirmed in approximately two thirds of the patients (65.2%).

**Table 6:** Histology of 233 PAR specimens^a^.

	PAR (*n*=233)	PAR-SMA (*n*=63)	PAR-CT/HA (*n*=138)	PAR-SMA+CT/HA (*n*=32)	*P*
Malignant tumor histology, *n* (%)	230 (98.7)	62 (98.4)	136 (98.6)	32 (100)	1.000
PDAC, *n* (%)	180 (78.2)	51 (81.0)	102 (73.9)	27 (84.4)	0.372
Malignant IPMN, *n* (%)	24 (10.2)	6 (9.5)	14 (10.1)	4 (12.5)	0.906
Cholangiocarcinoma, *n* (%)	6 (2.5)	1 (1.6)	5 (3.6)	0 (0)	0.596
Duodenal adenocarcinoma, *n* (%)	2 (0.8)	0 (0)	2 (1.5)	0 (0)	1.000
Pancreatic neuroendocrine tumor/carcinoma, *n* (%)	5 (2.1)	0 (0)	4 (2.9)	1 (3.1)	0.416
Sarcoma, *n* (%)	4 (1.7)	2 (3.2)	2 (1.5)	0 (0)	0.615
Pancreatic metastasis, *n* (%)	2 (0.8)	0 (0)	2 (1.5)	0 (0)	1.000
Other malignant tumor types, *n* (%)	7 (3.0)	2 (3.2)	5 (3.6)	0 (0)	0.751
Margins (malignant tumors)					
R0 resection, *n* (%)	163 (70.8)	49 (79.0)	92 (67.7)	22 (68.8)	0.252
R1 resection, *n* (%)	67 (29.1)	13 (21.0)	44 (32.4)	10 (31.3)	0.252
R2 resection, *n* (%)	0	0	0	0	NA
Site of R1 resection (malignant tumors)					
Anterior margin, *n* (%)^b^	26 (11.3)	5 (8.2)	14 (10.6)	7 (21.9)	0.151
Posterior margin, *n* (%)^b^	22 (9.5)	3 (4.9)	17 (11.9)	2 (6.3)	0.211
Vein margin, *n* (%)^b^	14 (6.0)	3 (4.9)	10 (7.6)	1 (3.1)	0.791
SMA margin, *n* (%)^b^	16 (7.0)	1 (1.6)	14 (10.6)	1 (3.1)	0.056
Pancreatic neck margin, *n* (%)^b^	2 (0.9)	0 (0)	1 (0.8)	1 (3.1)	0.337
Common bile duct margin, *n* (%)^b^	0 (0)	0 (0)	0 (0)	0 (0)	NA
Proximal duodenum margin, *n* (%)^b^	1 (0.4)	0 (0)	1 (0.8)	0 (0)	1.000
Mesenteric root, margin *n* (%)^b^	1 (0.4)	1 (1.6)	0 (0)	0 (0)	0.409
Multiple R1 margins (malignant tumors), *n* (%)^b^	19 (8.3)	1 (1.6)	16 (12.1)	1 (3.1)	0.0226
Lymph nodes (malignant tumors)					
Examined lymph nodes, median (IQR)	59 (41–80.8)	63.5 (50–90.3)	54 (36–76)	68 (51.3–89.8)	0.0083
Positive lymph nodes, *n* (%)	3 (1–7)	2 (1–7)	3 (1–7)	3 (2–10.3)	0.356
Vascular histology (malignant tumors)					
Positive vascular histology, *n* (%)	150 (65.2)	46 (74.2)	82 (60.3)	22 (68.8)	0.147
Artery histology (malignant tumors)					
Positive arterial histology, *n* (%)	83 (35.6)	20 (32.3)	48 (35.3)	15 (46.9)	0.360
Positive SMA histology, *n* (%)	28 (29.8)	20 (32.3)	NA	8 (25.0)	0.635
Positive CT/HA histology, *n* (%)	57 (33.9)	NA	48 (35.3)	9 (28.1)	0.536
Length of arterial infiltration, median (IQR), mm	10 (6.3–15)	10 (5–25)	10 (5.8–15)	10 (7.5–10)	0.519
Vein histology (malignant tumors)					
Positive vein histology, *n* (%)	113 (49.1)	45 (75)	53 (56.4)	15 (48.4)	0.0196
Length of vein infiltration, median (IQR), mm	15 (10–25)	17.5 (10.8–33.8)	5 (5–25)	15 (10–20)	0.130

a Three patients excluded due to segmental resection and reconstruction of the splenic artery.

b Data missing for the first five patients.

IPMN, intraductal papillary mucinuous tumor.

### Histology of PAR for PDAC

**Figure 5:**
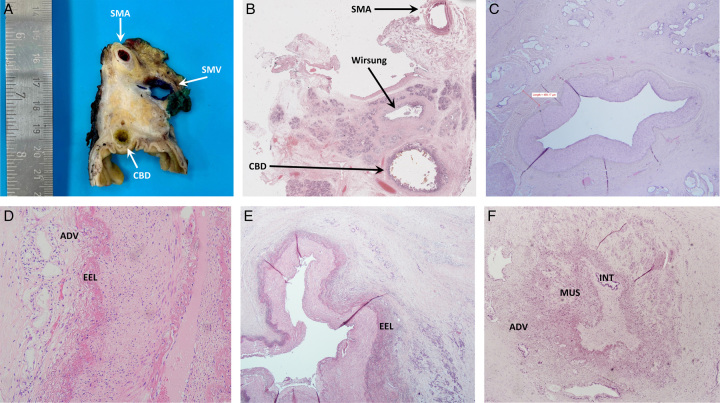
A pancreatectomy specimen in 3 mm-thick slices along the axial plane perpendicular to the duodenal axis, showing the superior mesenteric artery (SMA), superior mesenteric vein (SMV) and common bile duct (CBD). B Whole mount histology macrosection stained with hematoxylin and eosin, showing the superior mesenteric artery (SMA), Wirsung duct (Wirsung) and common bile duct (CBD). C Hepatic artery histology (hematoxylin-eosin staining, original magnification: 2×). The artery shows no tumor infiltration but the distance between the external elastic lamina and tumor is only 488.17 micron. In this case, arterial divestment could result in a microscopically positive margin (i.e. R1 resection). D Superior mesenteric artery histology (hematoxylin-eosin staining, original magnification: 2×). The tumor invades the tunica adventitia (ADV), while sparing the external elastic lamina (EEL). E Superior mesenteric artery histology (hematoxylin-eosin staining, original magnification: 2×). The tumor invades the external elastic lamina (EEL). F Hepatic artery histology (hematoxylin-eosin staining, original magnification: 2×). The vessel is surrounded by the tumor, which causes lumen reduction and invades the adventitial (ADV), muscular (MUS), and intimal (INT) layers.

The histological results of PAR-SMA and PAR-CT/HA for PDAC are presented in Table 7. Examples of histology studies are reported in Figure 5. PDAC was the final diagnosis in 180 PAR (77.5%). R1 resection was performed in 59 patients (32.7%): 20 (32.4%) following upfront resection and 39 (34.1%) after primary chemotherapy. Fifteen patients (8.3%) had multiple positive margin sites (Table 8).

**Table 7:** Histology of 180 PAR for PDAC^a^.

	PAR (*n*=180)	PAR-SMA (*n*=51)	PAR-CT/HA (*n*=102)	PAR-SMA+CT/HA (*n*=27)	*P*
Margins					
R0 resection, *n* (%)	121 (67.2)	39 (76.5)	64 (62.8)	18 (66.7)	0.233
R1 resection, *n* (%)	59 (32.7)	12 (23.5)	38 (37.3)	9 (33.3)	0.233
R2 resection, *n* (%)	0	0	0	0	NA
Site of R1 resection					
Anterior margin, *n* (%)^b^	23 (12.7)	4 (8)	13 (13.3)	6 (22.2)	0.210
Posterior margin, *n* (%)^b^	17 (9.4)	3 (6)	13 (13.3)	1 (3.7)	0.269
Vein margin, *n* (%)^b^	11 (6.1)	3 (6)	7 (7.1)	1 (3.7)	1.000
SMA margin, *n* (%)^b^	15 (8.4)	1 (2)	13 (13.3)	1 (3.7)	0.0498
Pancreatic neck margin, *n* (%)^b^	2 (1.1)	0 (0)	1 (1)	1 (3.7)	0.366
Common bile duct margin, *n* (%)^b^	0	0	0	0	NA
Proximal duodenum margin, *n* (%)^b^	1 (0.6)	0 (0)	1 (1)	0 (0)	1.000
Mesenteric root, margin *n* (%)^b^	1 (0.6)	1 (2)	0 (0)	0 (0)	0.433
Multiple R1 margins, *n* (%)^b^	15 (8.3)	1 (2)	13 (13.3)	1 (3.7)	0.0498
Lymph nodes					
Examined lymph nodes, median (IQR)	60.5 (41.3–83)	66 (54–91)	52.5 (36–79.8)	69 (51–89)	0.0097
Positive lymph nodes, *n* (%)	3 (1–7)	2 (1–7)	3 (1–7)	4 (2–11)	0.0839
Vascular histology					
Positive vascular histology, *n* (%)	122 (67.7)	39 (76.5)	65 (63.7)	18 (66.7)	0.280
Artery histology					
Positive arterial histology, *n* (%)	72 (39.3)	17 (33.3)	42 (41.2)	13 (48.2)	0.417
Positive SMA histology, *n* (%)	23 (29.5)	17 (33.3)	—	6 (22.2)	0.306
Positive CT/HA histology, *n* (%)	51 (39.5)	—	42 (41.2)	9 (33.3)	0.459
Length of arterial infiltration, median (IQR), mm	10 (6.3–15)	12.5 (5–27.5)	10 (6–15)	10 (7.5–10)	0.514
Vein histology					
Positive vein histology, *n* (%)	89 (49.4)	38 (74.5)	39 (30.2)	12 (44.4)	0.0108
Positive superior mesenteric vein/portal vein histology, *n* (%)	88 (48.8)	38 (74.5)	39 (38.2)	11 (34.3)	0.0055
Length of vein infiltration, median (IQR), mm	15 (10–25)	17.5 (10.8–33.8)	15 (9.3–25)	12 (10–19)	0.250

a Three patients excluded due to segmental resection and reconstruction of the splenic artery.

b Data missing for five patients.

**Table 8:** Sites of margin positivity in patients with PDAC histology.

	PRA	PAR-SMA	PAR-CT/HA	PAR-SMA+CT/HA	*P*
R1, *n* (%)	62 (33.9)	12 (23.5)	38 (37.3)	9 (33.3)	0.233
R1 anterior margin, *n* (%)	24 (13.5)	4 (8)	13 (13.3)	6 (22.2)	0.210
R1 posterior margin, *n* (%)	19 (10.7)	3 (6)	13 (13.3)	1 (3.7)	0.269
R1 vein margin, *n* (%)	12 (6.7)	3 (6)	7 (7.1)	1 (3.7)	1.000
R1 SMA margin, *n* (%)	15 (8.4)	1 (2)	13 (13.3)	1 (3.7)	0.0498
R1 neck of the pancreas margin, *n* (%)	2 (1.1)	0 (0)	1 (1)	1 (3.7)	0.366
R1 common bile duct margin, *n* (%)	0	0	0	0	NA
R1 proximal duodenum margin, *n* (%)	1 (0.6)	0 (0)	1 (1)	0 (0)	1.000
R1 mesenteric root, *n* (%)	1 (0.6)	1 (2)	0 (0)	0 (0)	0.433
Multiple R1 margins, *n* (%)	16 (9.0)	1 (2)	13 (13.3)	1 (3.7)	0.0498

### Long-term survival of PDAC

After a median follow-up period of 15.4 (8.8–28.3) months, 111 patients died and 55 were alive. Thirty-seven patients were alive and disease-free.

**Figure 6:**
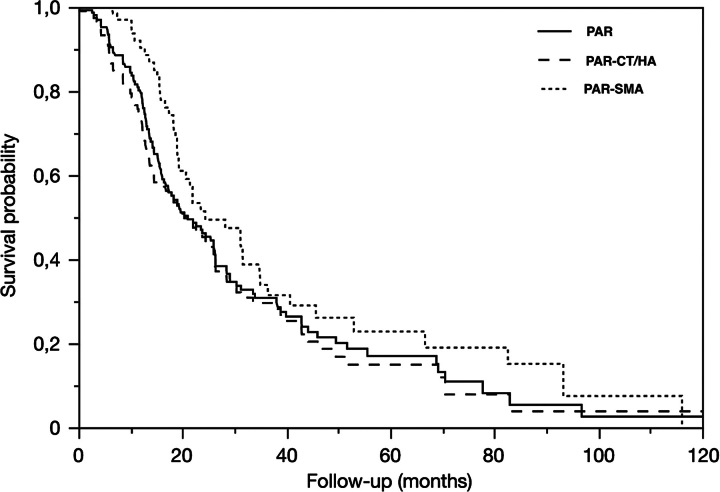
Kaplan–Meier survival curves (overall survival) of PAR (continuous line), PAR-CT/HA (long dash line), and PAR-SMA (dotted line) with pancreatic cancer histology results.

The median overall survival was 20.9 (12.5–42.8) months for PAR, 20.2 (14.4–44.0) months for PAR-SMA, and 20.2 (11.4–42.7) months for PAR-CT/HA (Fig. 6). Equivalent results by study decades were 12.0 (5.4–25.9) months, 15.1 (9.8–23.4) months, and 26.2 (14.3–51.5) months for 1993–2002, 2003–2012, and 2013–2023, respectively (*P*<0.0001).

### Sensitivity analysis of the impact of resection and reconstruction of the superior mesenteric artery on the occurrence of severe postoperative complications, postoperative mortality, and long-term survival

Supplementary Tables 1–4 (Supplemental Digital Content 5, http://links.lww.com/JS9/B558, Supplemental Digital Content 6, http://links.lww.com/JS9/B559, Supplemental Digital Content 7, http://links.lww.com/JS9/B560, Supplemental Digital Content 8, http://links.lww.com/JS9/B561) show the effect of potential confounders on the incidence of severe postoperative complications, postoperative mortality, and long-term survival. The only factor that affected postoperative mortality was prior abdominal surgery (OR=0.31, *P*=0.0437; Supplementary Table 2, Supplemental Digital Content 6, http://links.lww.com/JS9/B559). Long-term survival was predicted by median age [hazard ratio (HR)=1.03, *P*=0.0082], median ASA score (HR=0.59, *P*=0.0014), and history of cardiac disease (HR=2.13, *P*=0.0284) (Supplementary Table 3, Supplemental Digital Content 7, http://links.lww.com/JS9/B560). None of these factors affected the relationship between PAR-SMA and any of the aforementioned outcome measures. Similarly, according to the corresponding models, no potential categorical or continuous factor that could influence the study outcomes was unassessed (Supplementary Table 4, Supplemental Digital Content 8, http://links.lww.com/JS9/B561).

## Discussion

This study reports one of the largest world series of PAR and provides novel information. First, PAR was feasible, even in cases of PAR-SMA or multiple vessel resection. Second, the mortality of PAR reduced following completion of the learning curve. Some complications, such as intestinal or hepatic ischemia, were specific to PAR. They occurred rarely but had a high mortality index. Third, overcoming the learning curve of PAR required ~100 procedures. Fourth, R0 resection (circumferential margins of 1 mm) was achieved in 70% of PAR. Fifth, PAR permitted long-term survival, thus underscoring the importance of patient selection and referral to expert centers. Sixth, PAR-SMA was not associated with worse oncologic outcomes compared with those of PAR-CT/HA. Seventh, diarrhea and nutritional impairment occurred in the first six postoperative months but could be managed. Subsequently, all the patients had good intestinal function. Eighth, during the last decade of this study, the prognosis of patients undergoing PAR for PDAC improved dramatically.

Feasibility of PAR-CT/HA has already been established^28–30^. The novelty of this study is the feasibility of PAR-SMA in 95 consecutive patients. This is by far the largest reported information on PAR-SMA worldwide^31,32^. As this study confirmed that no clear proof of the association of PAR-SMA with worse prognosis, compared with that of PAR-CT/HA, exists, addressing concerns about PAR-SMA feasibility is important. However, the technical difficulty of PAR-SMA largely refers to SMA involvement and should not be underestimated. The current selection of surgical candidates for PAR-SMA mainly revolves around the new concept of a prognosis-based resectability that does not consider anatomical factors^9^. In this paradigm shift, the game changer is the availability of effective chemotherapy^33^. Following the administration of modern preoperative treatments, resection of pancreatic cancer is now deemed convenient when Ca 19.9 levels are decreasing (preferably low) and patient’s performance is good in the absence of tumor progression. Ca 19.9 is not a prefect prognostic tool, but currently, it is the best surrogate marker for defining the extent of occult systemic disease in PDAC.

The historically high mortality rate, often exceeding 10% at 30 days, has been a major concern regarding the implementation of PAR^34^. In this study, the mortality rate after PAR reduced from 16 to 4.6% after 106 procedures. The mortality rate decreased from 24.3 to 5.2% after 37 PAR-SMA and from 10.3 to 5.4% after 78 PAR-CT/HA. Experience with PAR-CT/HA may have contributed to speeding up the learning curve of PAR-SMA. These results are consistent with recently reported data by some high-volume centers^35–37^. Conversely, as demonstrated by a recent collaborative study, mortality remains above 10% in cases where PAR experience is sporadic^38^. Therefore, among experienced surgeons, concerns about excessive mortality associated with PAR, including PAR-SMA, should no longer be considered an absolute contraindication to pancreatectomy for an otherwise resectable tumor based on the new concept of prognosis-based resectability^9^.

The learning curve of PAR is poorly defined. Only one previous study addressed this issue and reported that 15 PAR were required to reduce mortality rate below 10%^37^. In this study 90-day mortality reduced below 5% after 106 PAR. The high number of PAR required to achieve a ‘reasonable’ mortality rate should not discourage pancreatic surgeons from performing these procedures. Experience with robotic pancreatoduodenectomy showed that the learning curve of newer generations of surgeons can be reduced dramatically^39^.

Despite the complexity of vascular reconstructions, the rate of vascular thrombosis reported in this study (5.5%) is consistent with data from the literature. In PAR, thrombosis incidence ranged from 4.2^35^ to 18%^40^. In benchmark patients undergoing pancreatoduodenectomy with venous resection, the overall rate of portal vein thrombosis and rate of occlusive thrombosis are expected to be less than or equal to 14% and less than or equal to 4%, respectively^41^. Technical refinements and tailored anticoagulant prophylaxis are both needed to improve these results. Since patients with established visceral ischemia are difficult to rescue, we have described an aggressive protocol of postoperative surveillance.

This study also showed that experience improved other relevant outcome metrics such as need for total gastrectomy, length of hospital and ICU stays, incidence of postoperative complications, overall burden of postoperative complications (i.e. comprehensive complication index), and incidence of arterial thrombosis.

Further important information from this study is the possibility to achieve high rates of R0 resection despite the locally advanced tumor stage. This result was achieved based on a highly standardized pathology protocol, as shown by the high number of examined lymph nodes. Interestingly, according to the concept of prognosis-based resectability, R0 rates are higher for PAR than for recent series of anatomically resectable PADC resected with upfront surgery^42^. These results also compare with those of previous studies on PAR. The R0 rate was 24% in Loos *et al.*’s^37^ study and ranged from 50% (pancreatoduodenectomy) to 61% (total pancreatectomy) in Bachellier *et al.*’s study^35^. In the current study, the relatively high R0 rates could reflect the extensive use of induction chemotherapy but could also mirror our standardized approach to PAR based on en-bloc resection of involved vascular segments.

This study also showed that PAR permits long-term survival despite the locally advanced tumor stage. Therefore, the main and yet largely unsolved problem is the appropriate selection of patients^12^. Patients who underwent surgery in the last decade experienced improved prognosis. Based on this result, we hope that improved understanding of tumor biology and more effective medical treatments can further enhance the oncologic value of PAR.

This study confirmed that all patients undergoing PAR suffered diarrhea, caused by autonomic denervation of the mid-gut, typically resulting in malnutrition. If no intervention is provided, the patient’s disease status worsens. If accurately managed, patients regain near-normal bowel function within the first six postoperative months.

Finally, during each of the three decades in this study, postoperative mortality decreased and long-term prognosis improved in patients diagnosed with PDAC. Considering the most recent decade, the 90-day postoperative mortality was 4.6% and median survival was 26.2 months. These results compare with either the mortality of contemporary benchmark pancreatoduodenectomy with vein resection^41^ or prognosis of patients with locally advanced PDAC treated using oncological therapies alone^43^.

In this study, all but 8 PAR (2.9%) were performed using the open approach. Although in rare circumstances and in extremely well selected patients, minimally invasive surgery for PAR may be feasible, an open approach seems to be most logical and efficient approach to dealing with complex procedures. Currently available guidelines do not encourage minimally invasive surgery if arterial resection is required^44,45^.

This study has several limitations. First, it is a retrospective study reporting on a 30-year experience; therefore, it is exposed to the bias of retrospective studies. Second, during this extended study period, the biology of PDAC was clarified, quality of imaging studies was improved, and effective oncologic treatments were highlighted. Therefore, the oncologic value of PAR cannot be defined based on the overall experience reported herein. The value should rather refer to patients who underwent surgery in the last decade. Third, this is not an intention-to-treat study. Therefore, the study misses some important information about the number of patients deemed unresectable based on preoperative imaging studies and about those who refused surgery. As previously stated, no resection was aborted due to technical reasons alone, but it was sometimes avoided based on preoperative features such as evidence of unreconstructible vessels. Fourth, PAR is technically demanding. The fact that some hepato-biliary-pancreatic surgeons, historically involved in transplantation and with sound background in vascular surgery, experience the challenges of PAR does not imply that similar results could be duplicated among average pancreatic or general surgeon. Therefore, generalizability of the results presented herein remains to be established. Fifth, this study lacks information about intraoperative medical management by the anesthesia team, which is primarily important for postoperative outcomes.

This study has some strengths. First, it describes the outcomes of a large series of PAR performed at a single center and provides cutoffs for completion of the learning curve regarding several key outcome metrics. Second, it describes the outcomes of the largest series of PAR-SMA ever reported. Third, it reports data on intestinal function following extensive retroperitoneal dissection, showing that consequences of intestinal denervation can be managed. Fourth, it demonstrates that postoperative mortality can be reduced over time, while long-term survival can be improved by induction chemotherapy and careful patient selection.

Further research should focus on improvements in patient selection, identification of the criteria for centralization specific to PAR, and definition of training pathways that enable more surgeons to perform PAR and possibly reduce the learning curve. Furthermore, the oncologic value of PAR should be assessed in a prospective, multicenter, randomized study.

## Conclusions

This study showed that PAR, including PAR-SMA, is feasible, and is currently associated with acceptable postoperative outcomes. Despite the long and steep learning curve, PAR results are improving and could be further refined in the future. Pancreatic surgeons should be prepared to address the technical challenges of PAR.

## Ethical approval

This study was approved by the Institutional Ethics Board of the University of Pisa (21060_Boggi).

## Consent

Written informed consent was obtained from the patient for publication and any accompanying images. A copy of the written consent is available for review by the Editor-in-Chief of this journal on request.

## Sources of funding

This study received no funds.

## Author contribution

N.N.: substantial contributions to conception and design, and acquisition of data, and analysis and interpretation of data for the work; revising the manuscript critically for important intellectual content; final approval of the version to be published; agreement to be accountable for all aspects of the work in ensuring that questions related to the accuracy or integrity of any part of the work are appropriately investigated and resolved. E.F.K.: acquisition of data; revising the manuscript critically for important intellectual content; final approval of the version to be published; agreement to be accountable for all aspects of the work in ensuring that questions related to the accuracy or integrity of any part of the work are appropriately investigated and resolved. M.G.: acquisition of data; revising the manuscript critically for important intellectual content; final approval of the version to be published; agreement to be accountable for all aspects of the work in ensuring that questions related to the accuracy or integrity of any part of the work are appropriately investigated and resolved. A.D.D.: acquisition of data; revising the manuscript critically for important intellectual content; final approval of the version to be published; agreement to be accountable for all aspects of the work in ensuring that questions related to the accuracy or integrity of any part of the work are appropriately investigated and resolved. L.L.: acquisition of data; revising the manuscript critically for important intellectual content; final approval of the version to be published; agreement to be accountable for all aspects of the work in ensuring that questions related to the accuracy or integrity of any part of the work are appropriately investigated and resolved. E.A.: acquisition of data; revising the manuscript critically for important intellectual content; final approval of the version to be published; agreement to be accountable for all aspects of the work in ensuring that questions related to the accuracy or integrity of any part of the work are appropriately investigated and resolved. F.V.: acquisition of data; revising the manuscript critically for important intellectual content; final approval of the version to be published; agreement to be accountable for all aspects of the work in ensuring that questions related to the accuracy or integrity of any part of the work are appropriately investigated and resolved. D.C.: acquisition of data; revising the manuscript critically for important intellectual content; final approval of the version to be published; agreement to be accountable for all aspects of the work in ensuring that questions related to the accuracy or integrity of any part of the work are appropriately investigated and resolved. C.C.: acquisition of data; revising the manuscript critically for important intellectual content; final approval of the version to be published; agreement to be accountable for all aspects of the work in ensuring that questions related to the accuracy or integrity of any part of the work are appropriately investigated and resolved. G.A.: acquisition of data; revising the manuscript critically for important intellectual content; final approval of the version to be published; agreement to be accountable for all aspects of the work in ensuring that questions related to the accuracy or integrity of any part of the work are appropriately investigated and resolved. U.B.: study conception and design, interpretation of data for the work; drafting the work and revising it critically for important intellectual content; final approval of the version to be published; agreement to be accountable for all aspects of the work in ensuring that questions related to the accuracy or integrity of any part of the work are appropriately investigated and resolved.

## Conflicts of interest discloure

The authors declare that they have no financial conflicts of interest with regard to the content of this report.

## Research registration unique identifying number (UIN)

1. Name of the registry: not applicable.
2. Unique identifying number or registration ID: not applicable.
3. Hyperlink to your specific registration (must be publicly accessible and will be checked): not applicable.

## Guarantor

Ugo Boggi.

## Data availability statement

Data are stored in an Institutional database. Data are available upon reasonable request to the senior author (responsible for correspondence about the manuscript).

## Provenance and peer review

Not commissioned, externally peer-reviewed.

## Supplementary Material

SUPPLEMENTARY MATERIAL
