# Supplementary material for: Postoperative results, learning curve, and outcomes of pancreatectomy with arterial resection: a single-center retrospective cohort study on 236 procedures
Source: Int J Surg. 2023 Dec 11;110(10):6111–25. doi: 10.1097/JS9.0000000000000971 (PMC11486960; doi:10.1097/JS9.0000000000000971)
Supplement: SUPPLEMENTARY MATERIAL [file js9-110-6111-s005.docx]

| **Supplementary Table 1.** Assessment of possible confounding factors for the occurrence of severe postoperative complications after PAR-SMA. | | |
| --- | --- | --- |
|  | **Univariate** | |
|  | **OR (IQR)** | **p** |
| PAR-SMA, n (%) | 1.20 (0.65-2.21) | 0.560 |
|  |  |  |
| *Possible confounding factors* |  |  |
| *Pre-operative factors* |  |  |
| Age, median (IQR), years | 1.006 (0.97-1.04) | 0.675 |
| Male gender, n (%) | 1.11 (0.61-2.04) | 0.734 |
| BMI, median (IQR), Kg/m2 | 0.94 (0.86-1.03) | 0.161 |
| ASA score, median (IQR) | 1.24 (0.77-1.97) | 0.378 |
| Diabetes, n (%) | 0.95 (0.47-1.89) | 0.875 |
| Cardiac disease, n (%) | 1.50 (0.58-3.87) | 0.397 |
| Chronic obstructive pulmonary disease, n (%) | 1.70 (0.49-5.86) | 0.404 |
| Previous abdominal surgery, n (%) | 0.67 (0.36-1.27) | 0.221 |
|  |  |  |
| *Intra-operative factors* |  |  |
| Pancreaticoduodenectomy, n (%) | 1.17 (0.51-2.67) | 0.715 |
| Total pancreatectomy, n (%) | 1.19 (0.61-2.34) | 0.604 |
| Distal pancreatectomy, n (%) | 0.62 (0.24-1.57) | 0.310 |
|  |  |  |
| *Pathological factors* |  |  |
| PDAC, n (%) | 0.92 (0.45-1.87) | 0.811 |
