# Supplementary material for: Postoperative results, learning curve, and outcomes of pancreatectomy with arterial resection: a single-center retrospective cohort study on 236 procedures
Source: Int J Surg. 2023 Dec 11;110(10):6111–25. doi: 10.1097/JS9.0000000000000971 (PMC11486960; doi:10.1097/JS9.0000000000000971)
Supplement: SUPPLEMENTARY MATERIAL [file js9-110-6111-s006.docx]

| **Supplementary Table 2.** Assessment of possible confounding factors for the occurrence of postoperative mortality after PAR-SMA. | | | | |
| --- | --- | --- | --- | --- |
|  | **Univariate** | | **Multivariate** | |
|  | **OR (IQR)** | **p** | **OR (IQR)** | **p** |
| SMA resection, n (%) | 1.71 (0.72-4.05) | 0.224 | 1.33 (0.55-3.24) | 0.528 |
|  |  |  |  |  |
| *Pre-operative factors* |  |  |  |  |
| Age, median (IQR), years | 1.01 (0.97-1.06) | 0.554 |  |  |
| Male gender, n (%) | 0.98 (0.42-2.33) | 0.970 |  |  |
| BMI, median (IQR), Kg/m2 | 1.02 (0.90-1.15) | 0.807 |  |  |
| ASA score, median (IQR) | 0.77 (0.40-1.47) | 0.421 |  |  |
| Diabetes, n (%) | 0.76 (0.27-2.14) | 0.604 |  |  |
| Cardiac disease, n (%) | 2.15 (0.67-6.97) | 0.202 |  |  |
| Chronic obstructive pulmonary disease, n (%) | 1.93 (0.40-9.42) | 0.414 |  |  |
| **Previous abdominal surgery, n (%)** | **0.29 (0.09-0.87)** | **0.0281** | **0.31 (0.09-0.97)** | **0.0437** |
|  |  |  |  |  |
| *Intra-operative factors* |  |  |  |  |
| Pancreaticoduodenectomy, n (%) | 0.85 (0.24-3.02) | 0.800 |  |  |
| Total pancreatectomy, n (%) | 2.18 (0.71-6.66) | 0.171 |  |  |
| Distal pancreatectomy, n (%) | 0.23 (0.03-1.77) | 0.159 |  |  |
|  |  |  |  |  |
| *Pathological factors* |  |  |  |  |
| PDAC, n (%) | 0.80 (0.30-2.15) | 0.661 |  |  |
