# Supplementary material for: Postoperative results, learning curve, and outcomes of pancreatectomy with arterial resection: a single-center retrospective cohort study on 236 procedures
Source: Int J Surg. 2023 Dec 11;110(10):6111–25. doi: 10.1097/JS9.0000000000000971 (PMC11486960; doi:10.1097/JS9.0000000000000971)
Supplement: SUPPLEMENTARY MATERIAL [file js9-110-6111-s007.docx]

| **Supplementary Table 3.** Assessment of possible confounding factors for long-term survival after PAR-SMA for ductal adenocarcinoma of the pancreas. | | | | |
| --- | --- | --- | --- | --- |
|  | **Univariate** | | **Multivariate** | |
|  | **HR (IQR)** | **p** | **HR (IQR)** | **p** |
| SMA resection, n (%) | 0.84 (0.57-1.23) | 0.367 | 0.95 (0.64-1.41) | 0.808 |
|  |  |  |  |  |
| *Pre-operative factors* |  |  |  |  |
| Age, median (IQR), years | **1.03 (1.009-1.05)** | **0.0049** | **1.03 (1.007-1.05)** | **0.0082** |
| Male gender, n (%) | 0.95 (0.65-1.38) | 0.774 |  |  |
| BMI, median (IQR), Kg/m2 | 0.97 (1.03-1.03) | 0.325 |  |  |
| ASA score, median (IQR) | **0.72 (0.54-0.97)** | **0.0294** | **0.59 (0.42-0.81)** | **0.0014** |
| Diabetes, n (%) | 0.89 (0.58-1.35) | 0.570 |  |  |
| Cardiac disease, n (%) | **1.81 (1.004-3.26)** | **0.0485** | **2.13 (1.08-4.20)** | **0.0284** |
| Chronic obstructive pulmonary disease, n (%) | 2.46 (0.99-6.13) | 0.0523 |  |  |
| Previous abdominal surgery, n (%) | 0.92 (0.63-1.36) | 0.688 |  |  |
|  |  |  |  |  |
| *Intra-operative factors* |  |  |  |  |
| Pancreaticoduodenectomy, n (%) | 1.53 (0.83-2.80) | 0.170 |  |  |
| Total pancreatectomy, n (%) | 0.76 (0.50-1.16) | 0.201 |  |  |
| Distal pancreatectomy, n (%) | 1.13 (0.69-1.84) | 0.623 |  |  |
