# Supplementary material for: Postoperative results, learning curve, and outcomes of pancreatectomy with arterial resection: a single-center retrospective cohort study on 236 procedures
Source: Int J Surg. 2023 Dec 11;110(10):6111–25. doi: 10.1097/JS9.0000000000000971 (PMC11486960; doi:10.1097/JS9.0000000000000971)
Supplement: SUPPLEMENTARY MATERIAL [file js9-110-6111-s008.docx]

| **Supplementary Table 4.** Association between PAR-SMA and the occurrence of severe post-operative complications, post-operative mortality, or long-term survival according to any unmeasured potential categorical or continuous variable. | | | | | | |  |
| --- | --- | --- | --- | --- | --- | --- | --- |
|  | | | | | | |  |
| **Severe Oostoperative Complications** | | | | | | |  |
|  | | | | | | |  |
|  |  |  | **Unmeasured potential categorical variable*** | | **Unmeasured potential continuous variable^** | | |
|  | **OR (IQR)** | **p** | **Adjusted OR (IQR)** | **p** | **Adjusted OR (IQR)** | **p** | |
|  |  |  |  |  |  |  | |
| PAR-SMA, n (%) | 1.20 (0.65-2.21) | 0.560 | 0.84 (0.45-1.54) | NS | 0.65 (0.35-1.21) | NS | |

*gamma= 2; p1= 1; ^gamma= 1.2, delta= 0.5 (*obsSens* R package)

| **Postoperative Mortality** | | | | | | |
| --- | --- | --- | --- | --- | --- | --- |
|  | | | | | | |
|  |  |  | **Unmeasured potential categorical variable*** | | **Unmeasured potential continuous variable^** | |
|  | **OR (IQR)** | **p** | **Adjusted OR (IQR)** | **p** | **Adjusted OR (IQR)** | **p** |
|  |  |  |  |  |  |  |
| PAR-SMA, n (%) | 1.20 (0.65-2.21) | 0.560 | 1.20 (0.49-2.96) | NS | 1.33 (0.54-3.29) | NS |
| **Previous abdominal surgery, n (%)** | **0.31 (0.09-0.97)** | **0.0437** |  |  |  |  |

*gamma= 2; p1= 1; ^gamma= 2, delta= 0.5 (*obsSens* R package)

| **Long-term Survival** | | | | | | |  |
| --- | --- | --- | --- | --- | --- | --- | --- |
|  | | | | | | |  |
|  |  |  | **Unmeasured potential categorical variable*** | | **Unmeasured potential continuous variable^** | | |
|  | **OR (IQR)** | **p** | **Adjusted OR (IQR)** | **p** | **Adjusted OR (IQR)** | **p** | |
|  |  |  |  |  |  |  | |
| PAR-SMA, n (%) | 0.95 (0.64-1.41) | 0.808 | 0.68 (0.46-1.007) | NS | 0.71 (0.47-1.04) | NS | |
| **Age, median (IQR), years** | **1.03 (1.007-1.05)** | **0.0082** |  |  |  |  | |
| **ASA score, median (IQR)** | **0.59 (0.42-0.81)** | **0.0014** |  |  |  |  | |
| **Cardiac disease, n (%)** | **2.13 (1.08-4.20)** | **0.0284** |  |  |  |  | |

*gamma= 2; p1= 0.4; ^gamma= 0.6, delta= 0.5 (*obsSens* R package)

|  |
| --- |
